# Self-reported dietary adherence, disease-specific symptoms, and quality of life are associated with healthcare provider follow-up in celiac disease

**DOI:** 10.1186/s12876-017-0713-7

**Published:** 2017-12-11

**Authors:** Jacob J. Hughey, Bonnie K. Ray, Anne R. Lee, Kristin N. Voorhees, Ciaran P. Kelly, Detlef Schuppan

**Affiliations:** 1 Department of Biomedical Informatics, Vanderbilt University School of Medicine, Nashville, TN USA; 2 Talkspace, New York, NY USA; 3 Celiac Disease Center, Columbia University Medical Center, New York, NY USA; 4 Beyond Celiac, Ambler, PA USA; 5 Division of Gastroenterology, Beth Israel Deaconess Medical Center, Boston, MA USA; 6 Institute of Translational Immunology, University Medical Center, Mainz, Germany

**Keywords:** Celiac disease, Diagnosis, Disease management, Genetic testing, Gluten-free diet, Healthcare provider, Patient-reported factors, Quality of life, Symptoms, Well-being

## Abstract

**Background:**

The only treatment for celiac disease (CeD) is a lifelong gluten-free diet (GFD). The restrictive nature of the GFD makes adherence a challenge. As an integral part of CeD management, multiple professional organizations recommend regular follow-up with a healthcare provider (HCP). Many CeD patients also participate in patient advocacy groups (PAGs) for education and support. Previous work found that follow-up of CeD patients is highly variable. Here we investigated the self-reported factors associated with HCP follow-up among individuals diagnosed with CeD who participate in a PAG.

**Methods:**

We conducted a survey of members of Beyond Celiac (a PAG), collecting responses from 1832 U.S. adults ages 19–65 who reported having CeD. The survey queried HCP follow-up related to CeD and included validated instruments for dietary adherence (CDAT), disease-specific symptoms (CSI), and quality of life (CD-QOL).

**Results:**

Overall, 27% of respondents diagnosed with CeD at least five years ago reported that they had not visited an HCP about CeD in the last five years. The most frequent reason for not visiting an HCP was “doing fine on my own” (47.6%). Using multiple logistic regression, we identified significant associations between whether a respondent reported visiting an HCP about CeD in the last five years and the scores for all three validated instruments. In particular, as disease-specific symptoms and quality of life worsened, the probability of having visited an HCP increased. Conversely, as dietary adherence worsened, the probability decreased.

**Conclusions:**

Our results suggest that many individuals with CeD manage their disease without ongoing support from an HCP. Our results thus emphasize the need for greater access to high quality CeD care, and highlight an opportunity for PAGs to bring together patients and HCPs to improve management of CeD.

**Electronic supplementary material:**

The online version of this article (10.1186/s12876-017-0713-7) contains supplementary material, which is available to authorized users.

## Background

Celiac disease (CeD) is a chronic condition with autoimmune features driven by dietary consumption of gluten (a group of proteins present in wheat, barley, and rye) in genetically susceptible individuals [1]. The prevalence of CeD is estimated to be approximately 1% in most Western countries, although many individuals with CeD remain undiagnosed [2, 3]. Currently, the only treatment for CeD is a strict, lifelong gluten-free diet (GFD). Although the prognosis for most CeD patients on the GFD is good, the ubiquity of gluten in the Western diet and the need to avoid even minor contamination of otherwise gluten-free foods make adherence to the GFD a severe challenge [4–6]. Adherence to the GFD is highly variable and many CeD patients report a treatment burden that is comparable to patients with end-stage renal disease on dialysis [7, 8].

As an integral part of CeD management, guidelines from the American Gastroenterological Association (AGA) and the American College of Gastroenterology (ACG) and a consensus statement from the U.S. National Institutes of Health recommend regular follow-up with a healthcare provider (HCP) [9–11]. Such follow-up with a doctor and/or dietitian is essential for providing accurate information about the GFD, improving adherence to the GFD, verifying normalization of serology and other abnormalities found during diagnosis, monitoring symptoms, and checking for complications. The NIH consensus statement also recommends participation in a patient advocacy group (PAG) as a means to improve dietary adherence and to obtain emotional and social support. Unfortunately, a previous study found that HCP follow-up for many CeD patients is inadequate by multiple measures [12]. Overall, only 35% of patients followed for more than four years after diagnosis received care consistent with AGA recommendations.

Although improved HCP follow-up could enable improved CeD management, the factors that influence variation in HCP follow-up in CeD remain unknown. Importantly, several improved questionnaires have now been developed and validated to assess various aspects of life with CeD [13–15]. To our knowledge, however, these questionnaires have not yet been applied to understand how HCP follow-up is related to factors such as an individual’s CeD-related symptoms and quality of life. The aim of this study was to identify the patient-specific factors associated with HCP follow-up among adults diagnosed with CeD who participate in the online network of a national PAG.

## Methods

### Survey design and distribution

The survey was designed by a committee of CeD physicians and researchers, as well as patient representatives from Beyond Celiac. The committee identified validated instruments to include and developed new questions to capture information regarding patients’ healthcare utilization as well as personal and family medical history. The survey was reviewed by individuals with CeD for readability, clarity, and comprehensiveness before being sent to participants.

The survey was divided into five categories: Demographics, Emotional Well-Being, Celiac Disease-Specific Health, Gluten-Free Diet, Natural Course of Celiac Disease and Celiac Disease Diagnosis and Management. The survey comprised a total of 51 questions, with 15 including multiple sub-questions. The total number of questions a participant was asked to answer was dependent on the participant’s age (12 years old and under, 13–18 years old, and 19 years old and above).

The study population consisted of individuals active in the online community of Beyond Celiac, a national PAG in the United States. Beyond Celiac disseminated announcements about the survey to over 49,000 people using email and shared the survey among a network of more than 114,000 Facebook followers from March 19 to March 31, 2015. The announcements included a description of the survey and a link to complete the survey.

For this paper, we analyzed data from respondents who were 19–65 years old, living in the United States, and personally diagnosed with CeD. Analysis of the survey data was approved as non-human subjects research by the Vanderbilt University IRB (#161349).

### Validated instruments

The survey included validated instruments for dietary adherence (CDAT), CeD-specific symptoms (CSI), and quality of life (CD-QOL) [13–15]. Higher CDAT and CSI scores correspond to worse dietary adherence and CeD-specific symptoms, respectively, whereas a higher CD-QOL score corresponds to better quality of life. Two questions are common to both the CDAT and CSI ("Have you been bothered by headaches during the past 4 weeks?" and "Have you been bothered by low energy levels during the past 4 weeks?"). These two questions were each asked only once in the survey and unless noted otherwise, were used to calculate the score for both instruments. One question in the CSI ("How much physical pain have you had during the past 4 weeks?") and one element in the CD-QOL ("I have trouble socializing because of my disease.") were inadvertently omitted from the survey.

All elements of the CD-QOL have negative valence, with one exception (“I feel the diet is sufficient treatment for my disease”). For consistency with previous work, we calculated the CD-QOL score as the sum of scores from the individual elements after first reversing the scores of elements with negative valence. For example, since the score for each element could go from 1 to 5, a score of 4 (“quite a bit”) for the element "I feel frightened by having this disease" was converted to a 2.

### Analysis and visualization

**Fig. 1:**
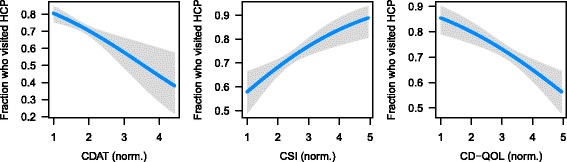
Visualization of multiple logistic regression model from Table 4. In each plot, the blue line shows the estimated fraction of respondents who visited an HCP in the last 5 y as a function of the score for the respective instrument (with the scores of the other two instruments and age held constant at their respective medians). Gray areas indicate 95% Wald confidence intervals

Analysis was performed in R 3.4.0 [16]. Venn diagrams were generated using the VennDiagram package [17]. For multiple logistic regression, we first divided the score of each instrument by the number of elements in the instrument, resulting in a scaled score that could range between 1 and 5. This has no effect on statistical significance and makes the coefficients easier to compare to each other. In addition, age group was converted to an integer value. Coefficients for the multiple logistic regression based on all three instruments (Fig. 1) were visualized using the visreg package [18]. For multiple logistic regression based on individual elements in each instrument, *p*-values were converted to q-values (controlling the false discovery rate) using the method of Benjamini and Hochberg [19].

## Results

The survey was made available to members of Beyond Celiac and responses were collected from 1832 U.S. adults ages 19–65 who reported having CeD. The survey was designed to query multiple aspects of life with CeD. We first examined the respondents’ demographics (Table 1). Overall, 89% of respondents were female, which is somewhat higher than previous studies [12–15]. The vast majority of respondents (95%) reported their race as “White or Caucasian,” which is similar to a previous study based on individuals from an academic medical center and celiac support groups [15] and which could be a result of the higher frequency of CeD in non-Hispanic whites than in Hispanics or non-Hispanic blacks [20]. Respondents came from every age group and region of the U.S.

**Table 1:** Demographics of survey respondents (*n* = 1832)

Gender	
Female	89.0%
Male	11.0%
Age (y)	
19–25	7.9%
26–35	18.8%
36–45	23.7%
46–55	26.0%
56–65	23.6%
Race/ethnicity	
White or Caucasian	95.2%
Hispanic or Latino	2.2%
Black or African American	0.7%
Asian or Pacific Islander	0.2%
Other^a^	1.6%
Region of the U.S.	
Midwest	23.6%
Northeast	30.0%
South	28.9%
West	17.6%

^a^Includes Native American or American Indian, Indian, and prefer not to say

We next examined how and by whom the respondents reported being diagnosed with CeD (Table 2). Overall, 83% of respondents were diagnosed by a pediatrician, primary care provider, or gastroenterologist (80% of respondents also considered one of these their main provider for CeD; Additional file 1: Figure S1). In addition, similar to previous work [15], 95% were diagnosed based on a blood test (83%), small intestinal biopsy (79%), or both (67%; Additional file 1: Figure S2). Although these distributions were similar in each age group, we did observe higher frequencies of blood test and gluten challenge in younger age groups and a lower frequency of small intestinal biopsy in the youngest age group (19–25 y.o.; Additional file 1: Figure S3). Respondents reported an array of other autoimmune or CeD-related conditions (Additional file 1: Figure S4), although the prevalence of type 1 diabetes was less than expected [21]. Taken together, these results suggest that the respondents are reasonably representative of U.S. adults diagnosed with CeD.

**Table 2:** Self-reported information on how and by whom respondents were diagnosed with CeD

Person who made diagnosis	
Gastroenterologist	57.0%
Primary care provider	24.1%
Pediatrician	2.0%
Dietitian or nutritionist	0.8%
Self-diagnosed	2.1%
Other	13.9%
Methods of diagnosis^a^	
Blood test	83.0%
Small intestinal biopsy	78.8%
Gluten challenge	14.8%
Genetic test (e.g., HLA)	14.1%
Not sure	1.9%

^a^Not mutually exclusive

We then analyzed the respondents’ reported HCP follow-up. Of 1493 respondents diagnosed at least five years ago, 27% reported that they had not visited an HCP about CeD in the last five years (Table 3). Although we do not have additional data (e.g., medical records) to evaluate the follow-up care received by those individuals who did report visiting an HCP, these results suggest that many U.S. adults diagnosed with CeD are managing their disease without ongoing support from an HCP. We next quantified the reasons that respondents gave for not visiting an HCP. The most frequent reason was “doing fine on my own” (47.6%), followed by “haven’t needed to,” “provider was not knowledgeable,” and “previous visits were not helpful” (Table 3, Additional file 1: Figure S5). Financial reasons (“co-pay is too high” and “uninsured”) and general distrust of HCPs were less common.

**Table 3:** Self-reported information on HCP follow-up among respondents diagnosed at least 5 y ago

Visited HCP in last 5 y	*n* = 1493
Yes	65.6%
No	27.1%
Not sure	7.4%
Reasons for not visiting HCP^a^	*n* = 479
Doing fine on my own	47.6%
Haven’t needed to	28.0%
Provider not knowledgeable	27.6%
Previous visits not helpful	23.6%
Co-pay is too high	7.9%
Don’t trust healthcare providers	5.0%
Uninsured	3.5%

^a^Not mutually exclusive

To better understand the factors related to HCP follow-up, we used the survey responses from the validated instruments for dietary adherence (CDAT), disease-specific symptoms (CSI), and quality of life (CD-QOL) (Additional file 1: Figure S6) [13–15]. Scores from all three instruments were significantly better in respondents diagnosed at least five years ago than in respondents diagnosed more recently (Additional file 1: Figure S7). For those respondents diagnosed at least five years ago, we then used their age group and CDAT, CSI, and CD-QOL scores in multiple logistic regression to predict whether an individual reported visiting an HCP about CeD in the last five years (Table 4). Scores from all three instruments, but not age group, were significantly associated with having visited an HCP. In particular, as celiac-specific symptoms and quality of life worsened (indicated by higher CSI and lower CD-QOL scores, respectively), the probability of having visited an HCP increased (Fig. 1). Conversely, as dietary adherence worsened (indicated by a higher CDAT score), the probability decreased. These results suggest that although scores from the three instruments are moderately correlated with each other (Additional file 1: Table S1), each instrument captures a unique aspect of life with CeD.

**Table 4:** Multiple logistic regression to predict whether respondent has visited an HCP about CeD in last 5 years (assuming he or she was diagnosed at least 5 years ago)

	Coefficient	Std. error	*P*-value
Age group	0.081	0.049	0.098
CDAT score	−0.56	0.16	3.6*10^−4^
CSI score	0.45	0.13	4.2*10^−4^
CD-QOL score	−0.38	0.096	5.8*10^−5^

Positive coefficient means that as age or score increases, probability of visiting an HCP increases. Coefficients and standard errors for CDAT, CSI, and CD-QOL are normalized by the number of questions in the instrument. A higher CD-QOL corresponds to better quality of life, whereas higher CDAT and CSI scores correspond to worse dietary adherence and disease-specific symptoms, respectively

**Fig. 2:**
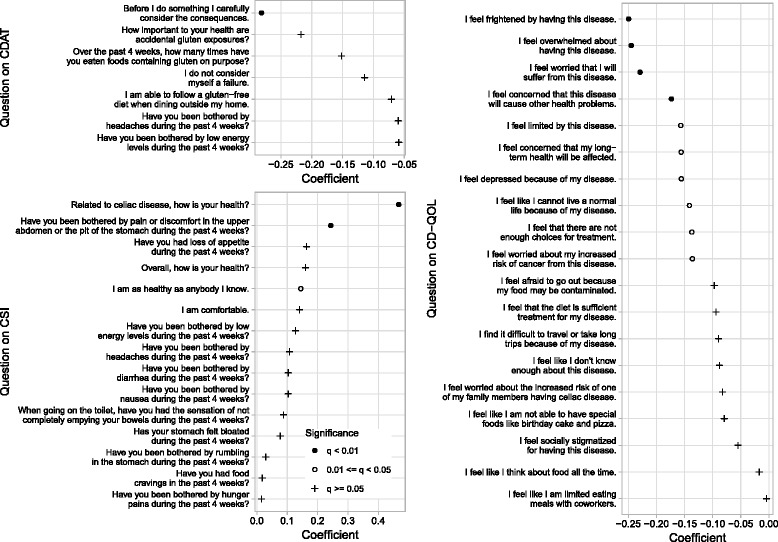
Coefficients and estimated FDR for association (in multiple logistic regression) between whether respondent has visited HCP in last 5 y and the response to individual questions in each instrument. *P*-values were converted to q-values to control for false discovery rate. Coefficients for questions in the CD-QOL were estimated after adjusting for valence, which means the scores of all individual questions except for one (“I feel the diet is sufficient treatment for my disease”) were reversed

Finally, we determined which individual questions or statements in each instrument were the most strongly associated with having visited an HCP. For each instrument, we performed multiple logistic regression using age group, scores from the other two instruments, and the response to one question of the selected instrument (Fig. 2). The three instruments varied in the fraction of questions or statements that were significantly associated with visiting an HCP. In each instrument, we observed the strongest associations with questions or statements that referred to more general aspects of life with CeD. In the CDAT, the strongest association was with the statement “Before I do something I carefully consider the consequences” (stronger agreement indicated higher probability of having visited an HCP). In the CSI, the strongest association was with the question “Related to celiac disease, how is your health?” (worse health indicated higher probability of having visited an HCP). In the CD-QOL, the strongest association was with the statement “I feel frightened by having this disease” (stronger agreement indicated higher probability of having visited an HCP).

## Discussion

Celiac disease is a complex condition with autoimmune characteristics for which the only current treatment is a strict, lifelong, gluten-free diet. Although recommendations for managing CeD include regular follow-up with an HCP, follow-up is often inadequate [12]. Here we studied the factors associated with patient-reported follow-up related to CeD in a large group of U.S. adults. Over a quarter of our survey respondents who were diagnosed at least five years ago reported not having visited an HCP about CeD in the last 5 years. In addition, individuals who have visited an HCP about CeD generally have better dietary adherence, worse symptoms, and worse quality of life than those who have not.

The survey question about HCP follow-up was designed to be broad and straightforward to answer. The limitation is that our metric for HCP follow-up is necessarily coarse and does not account for all the variation in the quantity, and more importantly the quality, of care. Thus, even for the 66% of respondents who reported having visited in HCP about CeD in the last five years, it is likely that the level of follow-up often does not meet AGA recommendations [12].

Individuals with CeD face high rates of underdiagnosis and misdiagnosis and often endure 5–10 years of symptoms before being correctly diagnosed [22–24]. Such a long and frustrating “diagnostic odyssey” could negatively influence patients’ opinions of the healthcare system and reduce the likelihood that they continue to interact with it after diagnosis. Notably, 37.8% of respondents to our survey reported not visiting an HCP in the last five years because their provider was not knowledgeable and/or previous visits were not helpful. If this is the case, then increasing the speed of an accurate diagnosis could contribute to improved CeD management after diagnosis.

To our knowledge, this is the first study to collect responses from the same individuals for three validated, CeD-related, instruments. Although the inadvertent omission of one element each from two of the instruments prevents strict comparison with previous studies, we reproduced the correlation between dietary adherence and symptoms [14] and between dietary adherence and quality of life [25]. Furthermore, our analysis allowed us to disentangle the effects of the three instruments on CeD-related follow-up. For example, even though better dietary adherence is positively correlated with better symptoms, a higher probability of visiting an HCP is associated with better adherence and worse symptoms. In addition, by analyzing the responses to individual elements of each instrument, we discovered that the correlates of having visited an HCP follow a hierarchy, at the top of which are more general concerns related to CeD, such as one’s overall perception of health related to CeD and feeling frightened by CeD.

We observed multiple trends in the frequency of diagnosis methods between age groups. Individuals aged 19–25 reported the lowest frequency of small intestinal biopsy, which may reflect the growing willingness of HCPs to diagnose CeD without a biopsy [26, 27]. For example, the most recent European Society for Pediatric Gastroenterology, Hepatology, and Nutrition (ESPGHAN) guidelines omit the need for a small intestinal biopsy, as long as IgA anti-transglutaminase (TG2) antibody titers are >10× the upper limit of normal and other clinical criteria are met [28]. We also found that younger age groups reported higher frequencies of gluten challenge. For example, 25% of respondents aged 19–25 reported that a gluten challenge was part of their diagnostic workup. Although our survey was not designed to address this issue, we speculate that this result may be related to the increasing prevalence of the GFD among people not diagnosed with CeD, especially among younger people [29]. Because the blood tests and small intestinal biopsy currently used for diagnosis are markers of active disease, a person who has been following a GFD will typically have to resume gluten consumption for 2–6 weeks (the gluten challenge) in order to be definitively diagnosed [11]. The gluten challenge is often accompanied by the return of symptoms, making this method of diagnosis burdensome and making patients reluctant to obtain a definitive diagnosis [30]. Notably, the need for a gluten challenge is obviated by a negative genetic test for HLA-DQ2 or -DQ8 [11], which excludes CeD, and we speculate that for most survey participants who underwent both genetic testing and a gluten challenge, the former was used to rule in (but not necessarily confirm) CeD. Our findings thus highlight the need for diagnostic tools that do not depend on ongoing gluten consumption.

Our study has several limitations. First, although our sample size is large, it is non-random and could suffer from self-selection bias based on who received notification of the survey (those who had previously interacted with the online community of Beyond Celiac) and who chose to respond. For example, involvement in support groups has been associated with less severe CeD-related symptoms [14] and involvement in face-to-face social support networks has been associated with higher CeD-related quality of life [31]. However, we observed considerable variation in the scores for all three instruments, which indicates that our respondents cover a wide range of lifestyles with respect to managing CeD. Second, our study is based only on self-reported data, and we do not have the respondents’ medical records to confirm their diagnosis or their follow-up with an HCP. However, the frequency of various diagnostic methods in our data is consistent with previous studies, which suggests that the self-reported data from our respondents is accurate. Moreover, the high level of self-management required of CeD patients (often before and after diagnosis) means that they are typically well informed about their disease and diagnostic workup.

A third limitation is that our study is cross-sectional and observational, so it is not possible to determine causality in the associations we observe. That said, we find it unlikely that visiting an HCP about CeD causes worse symptoms and quality of life. We believe the more likely scenario is that symptoms and quality of life influence how a person manages CeD, in particular, whether a person seeks support from an HCP. Individuals who are satisfied with or who can tolerate their symptoms and quality of life may be less likely to visit an HCP, possibly because they feel that the lack of non-dietary treatment options means that HCPs have little to offer. The association between dietary adherence and having visited an HCP also has multiple interpretations. One is that visiting an HCP causes improved adherence, perhaps because HCPs provide patients with a better understanding of how to implement the diet or a better appreciation of the diet’s importance. Another interpretation is that both dietary adherence and visits to an HCP are indirect measures for how conscientiously a patient manages his or her disease. These interpretations are not mutually exclusive, and it is likely that both are valid to varying extents among CeD patients.

## Conclusions

Our results suggest that many adults with CeD manage their disease without ongoing follow-up care from an HCP. This study also demonstrates the ability of PAGs to rapidly engage CeD patients for research, which is encouraging for future efforts using patient input to drive clinical research directed at a better understanding of cofactors that trigger CeD, improved disease management, and non-dietary therapies [32]. Finally, our results emphasize the need for greater access to high quality CeD care, and highlight an opportunity for HCPs and PAGs to work collaboratively to achieve improved disease management by raising levels of awareness and education.

## Additional file

Additional file 1: Supplemental Figures and Table.
**Figure S1.** Self-reported information on who the respondent considers his/her main provider for CeD. **Figure S2.** Venn diagram of self-reported diagnosis methods across all respondents. **Figure S3.** Self-reported information on methods of diagnosis for respondents in each age group. **Figure S4.** Self-reported prevalence of other autoimmune or related conditions among all respondents. **Figure S5.** Venn diagram of self-reported reasons for not visiting an HCP about CeD. **Figure S6.** Histograms of normalized scores for each instrument. **Figure S7.** Boxplots of normalized scores for each instrument vs. age and time since diagnosis. **Table S1.** Spearman correlation between scores of instruments. (PDF 693 kb)

## Abbreviations

ACG: American College of Gastroenterology
AGA: American Gastroenterological Association
CDAT: celiac dietary adherence test
CD-QOL: celiac disease-related quality of life
CeD: celiac disease
CSI: celiac symptom index
FDR: false discovery rate
GFD: gluten-free diet
HCP: healthcare provider
PAG: patient advocacy group
