# Supplementary material for: Self-reported dietary adherence, disease-specific symptoms, and quality of life are associated with healthcare provider follow-up in celiac disease
Source: BMC Gastroenterol. 2017 Dec 11;17:156. doi: 10.1186/s12876-017-0713-7 (PMC5725827; doi:10.1186/s12876-017-0713-7)
Supplement: Additional file 1: Supplemental Figures and Table. — Figure S1. Self-reported information on who the respondent considers his/her main provider for CeD. Figure S2. Venn diagram of self-reported diagnosis methods across all respondents. Figure S3. Self-reported information on methods of diagnosis for respondents in each age group. Figure S4. Self-reported prevalence of other autoimmune or related conditions among all respondents. Figure S5. Venn diagram of self-reported reasons for not visiting an HCP about CeD. Figure S6. Histograms of normalized scores for each instrument. Figure S7. Boxplots of normalized scores for each instrument vs. age and time since diagnosis. Table S1. Spearman correlation between scores of instruments. (PDF 693 kb) [file 12876_2017_713_MOESM1_ESM.pdf]

Supplementary Material for:

Self-reported dietary adherence, disease-specific symptoms, and quality of life are associated with healthcare provider follow-up in celiac disease

Jacob J. Hughey[1*], Bonnie K. Ray[2], Anne R. Lee[3], Kristin N. Voorhees[4], Ciaran P. Kelly[5], Detlef Schuppan[5,6]

[1]Department of Biomedical Informatics, Vanderbilt University School of Medicine, Nashville, TN. [2]Talkspace, New York, NY. [3]Celiac Disease Center, Columbia University Medical Center, New York, NY. [4]Beyond Celiac, Ambler, PA. [5]Division of Gastroenterology, Beth Israel Deaconess Medical Center, Boston, MA. [6]Institute of Translational Immunology, University Medical Center, Mainz, Germany.

* Correspondence: jakejhughey@gmail.com

Figure S1

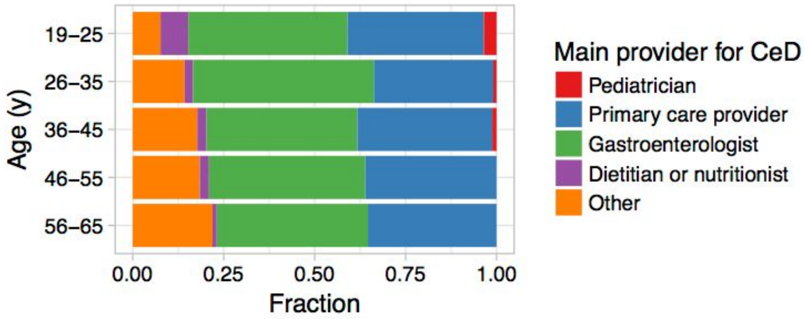

Self-reported information on who the respondent considers his/her main provider for CeD.

Figure S2

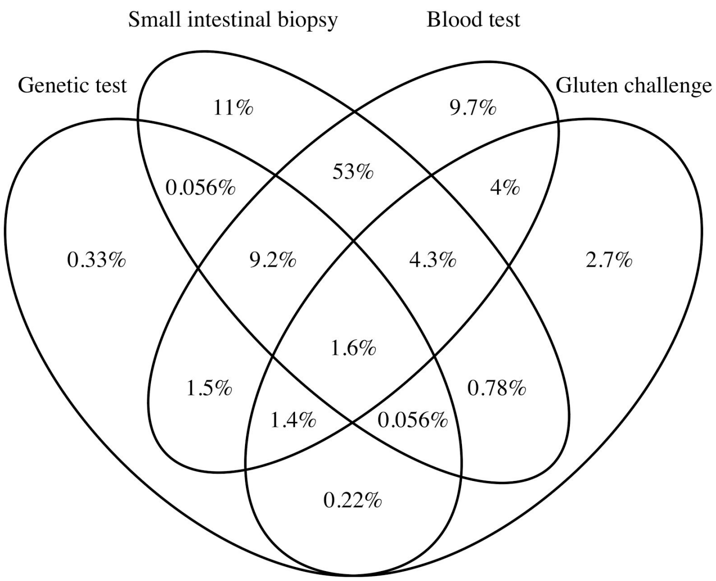

Venn diagram of self-reported diagnosis methods across all respondents.

Figure S3

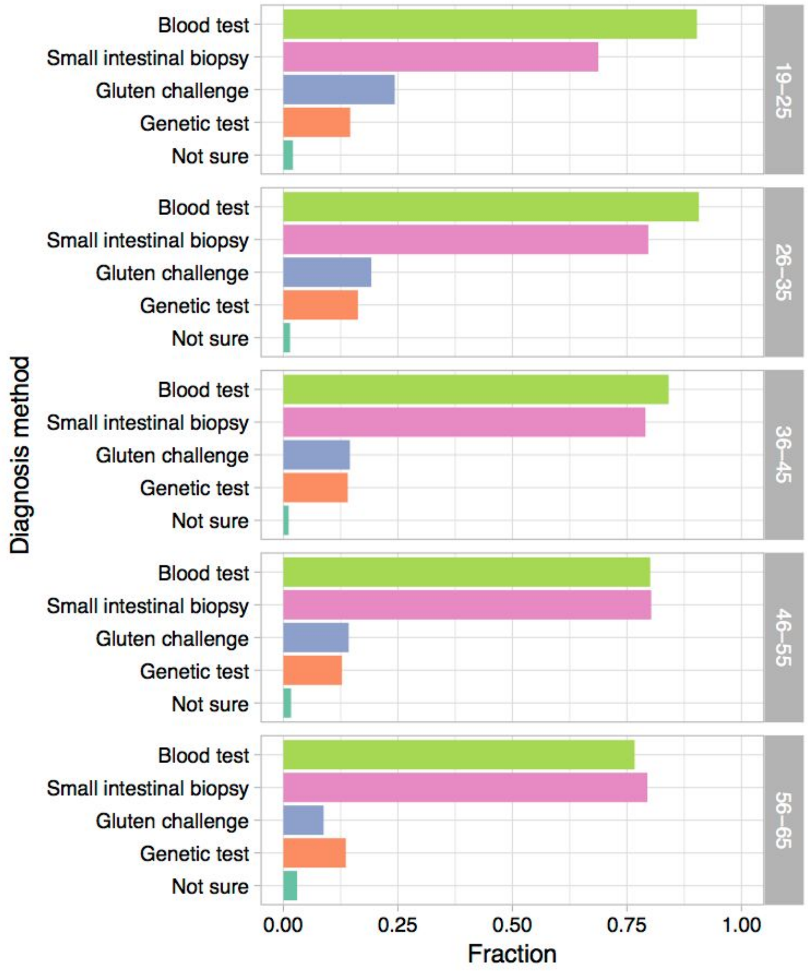

Self-reported information on methods of diagnosis for respondents in each age group. Based on a multi-group proportions test, there were significant differences between age groups in the frequency of blood test ($P = 3.8 * 10^{-7}$), small intestinal biopsy (P = 0.048), and gluten challenge ($P = 1.1 * 10^{-5}$).

Figure S4

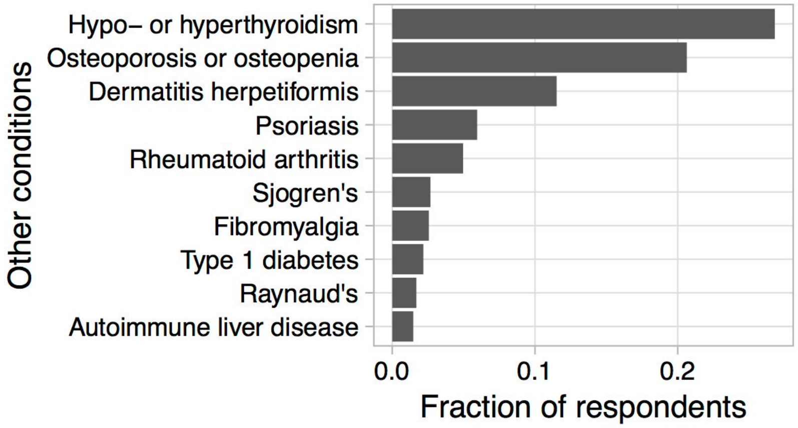

Self-reported prevalence of other autoimmune or related conditions among all respondents.

Figure S5

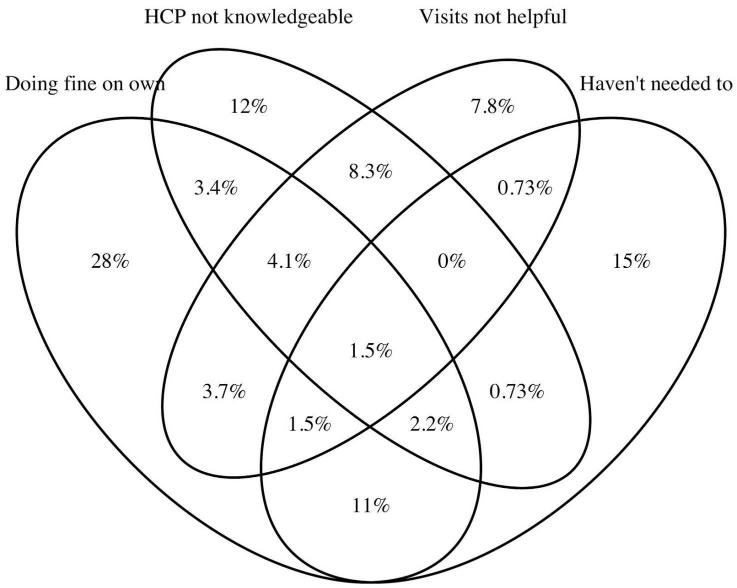

Venn diagram of self-reported reasons for not visiting an HCP about CeD. For ease of visualization, only the top 4 most commonly reported reasons are shown.

Figure S6

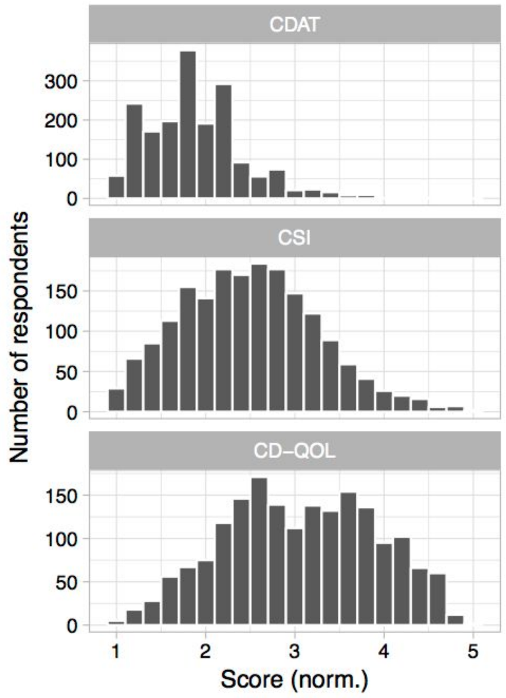

Histograms of normalized scores for each instrument. To normalize, the raw score was divided by the number of questions, so the normalized score corresponds to the average response (between 1 and 5).

Figure S7

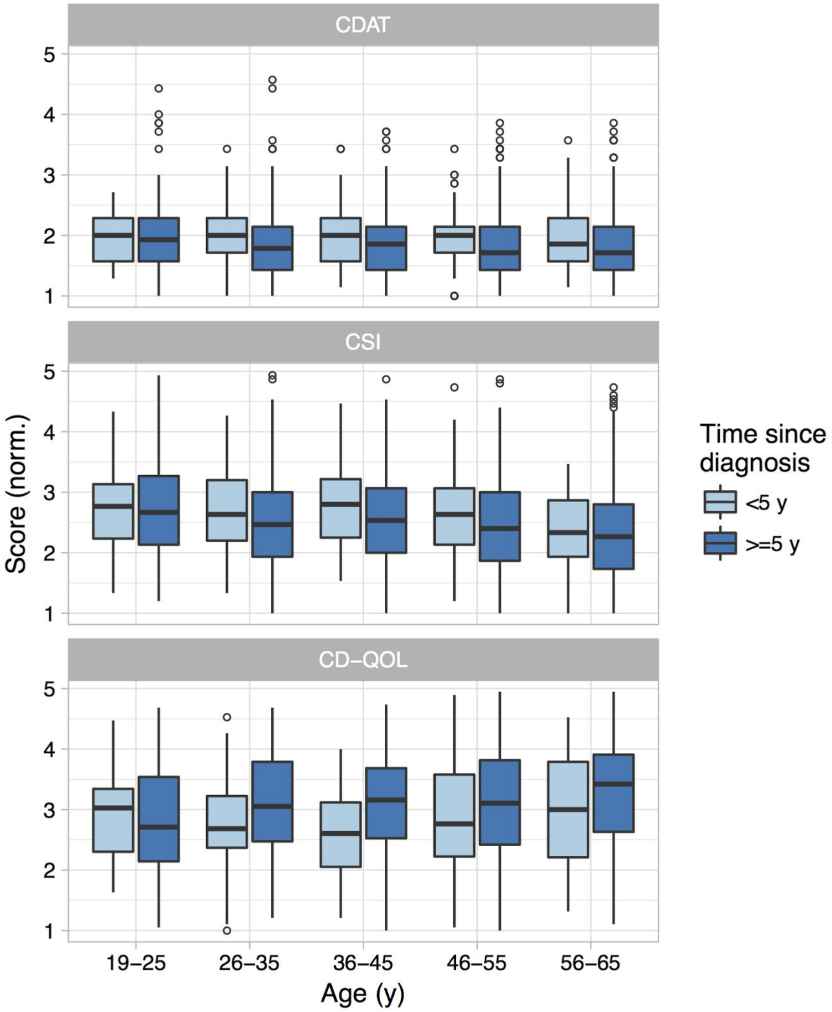

Boxplots of normalized scores for each instrument vs. age and time since diagnosis. Pooling data across age groups and using the Wilcoxon rank-sum test, scores for CDAT ($P=2.7*10^{-6}$), CSI ($P=1.8*10^{-6}$), and CD-QOL ($P=8.9*10^{-11}$) were significantly different between respondents diagnosed less than five years ago and respondents diagnosed at least five years ago.

Table S1

	CDAT	CSI	CSI (no overlap)	CD-QOL
CDAT	-	0.72	0.66	-0.54
CSI	0.72	-	0.99	-0.64
CSI (no overlap)	0.66	0.99	-	-0.63
CD-QOL	-0.54	-0.64	-0.63	-

Spearman correlation between scores of instruments. “CSI (no overlap)” refers to the set of questions in CSI minus the two questions that are also in CDAT. A higher CD-QOL corresponds to better quality of life, whereas higher CDAT and CSI scores correspond to worse dietary adherence and disease-specific symptoms, respectively.
